# Ecological Assessment of Autonomy in Instrumental Activities of Daily Living in Dementia Patients by the Means of an Automatic Video Monitoring System

**DOI:** 10.3389/fnagi.2015.00098

**Published:** 2015-06-02

**Authors:** Alexandra König, Carlos Fernando Crispim-Junior, Alvaro Gomez Uria Covella, Francois Bremond, Alexandre Derreumaux, Gregory Bensadoun, Renaud David, Frans Verhey, Pauline Aalten, Philippe Robert

**Affiliations:** ^1^EA CoBTeK, Université Côte d’Azur (UCA), Nice, France; ^2^Alzheimer Center Limburg, Maastricht University Medical Center, School for Mental Health and Neuroscience, Maastricht, Netherlands; ^3^STARS, INRIA, Sophia Antipolis, France; ^4^Centre Mémoire de Ressources et de Recherche, CHU de Nice, Nice, France

**Keywords:** dementia, Alzheimer, mild cognitive impairment, video analyses, assessment, information and communication technologies, autonomy, instrumental activities of daily living

## Abstract

Currently, the assessment of autonomy and functional ability involves clinical rating scales. However, scales are often limited in their ability to provide objective and sensitive information. By contrast, information and communication technologies may overcome these limitations by capturing more fully functional as well as cognitive disturbances associated with Alzheimer disease (AD). We investigated the quantitative assessment of autonomy in dementia patients based not only on gait analysis but also on the participant performance on instrumental activities of daily living (IADL) automatically recognized by a video event monitoring system (EMS). Three groups of participants (healthy controls, mild cognitive impairment, and AD patients) had to carry out a standardized scenario consisting of physical tasks (single and dual task) and several IADL such as preparing a pillbox or making a phone call while being recorded. After, video sensor data were processed by an EMS that automatically extracts kinematic parameters of the participants’ gait and recognizes their carried out activities. These parameters were then used for the assessment of the participants’ performance levels, here referred as autonomy. Autonomy assessment was approached as classification task using artificial intelligence methods that takes as input the parameters extracted by the EMS, here referred as behavioral profile. Activities were accurately recognized by the EMS with high precision. The most accurately recognized activities were “prepare medication” with 93% and “using phone” with 89% precision. The diagnostic group classifier obtained a precision of 73.46% when combining the analyses of physical tasks with IADL. In a further analysis, the created autonomy group classifier which obtained a precision of 83.67% when combining physical tasks and IADL. Results suggest that it is possible to quantitatively assess IADL functioning supported by an EMS and that even based on the extracted data the groups could be classified with high accuracy. This means that the use of such technologies may provide clinicians with diagnostic relevant information to improve autonomy assessment in real time decreasing observer biases.

## Introduction

One of the key features of Alzheimer’s disease (AD) is impairment in daily functioning as well as executive dysfunction due to global pathological changes in frontal and posterior areas (Marshall et al., 2006).

Studies show that in dementia patients, loss of functioning in instrumental activities of daily living (IADL) is strongly associated with faster cognitive decline (Arrighi et al., 2013) and, in particular, with poorer performances on executive function tasks (Razani et al., 2007; Karzmark et al., 2012) such as the frontal assessment battery (FAB) (Dubois et al., 2000) or the trail making test (version B) (Tombaugh, 2004). Hence, it represents an early predictor of cognitive deterioration and possibly even for conversion from mild cognitive impairment (MCI) to AD (Reppermund et al., 2013). This is in line with older findings that show that declines in IADL are influenced by cognitive functioning, and are affected relatively early in the course of dementia (Stern et al., 1990) and, in particular, the executive component in IADL tasks that requires higher frontal lobe activation (Baddeley et al., 1986).

The assessment of functioning in IADL has gradually attracted more attention in clinical research and should be included not only as a part of diagnostic evaluation in dementia but it would also be essential to evaluate efficacy in rehabilitation settings (Clare et al., 2003; Cotelli et al., 2006).

Characterizing impairment in IADL is controversial because no standard exists so far as to the practical or theoretical definition (DeBettignies et al., 1990). Furthermore, until now, the assessment of IADL has been mostly limited to questionnaires and relies often on informants’ reports, such as the disability assessment for dementia scale (DAD) or the IADL scale of Lawton and Brody (1969), which suffer from biases and inaccuracies in informants’ perceptions as well as the possibility that some older adults do not have an individual who can comment on their impact of cognitive impairment on routine activities. In general, existing functional assessments lack sufficient sensitivity to detect subtle functional changes or differences in behavior, and therefore, treatment effects (Gold, 2012). This leads to an urgent need for better measures of functional changes in people with the earliest changes associated with AD in clinical trials (Snyder et al., 2014).

Besides, just a few of the named tools capture the earliest functional deficits seen in preclinical AD.

Growing recognition of the need for a more objective and direct measurement has led to some attempts to improve the assessments of IADL in clinical practice by developing new extensive informant-based computerized IADL questionnaire (Sikkes et al., 2012) or direct performance-based measures (Moore et al., 2007), which differ from the traditional informant-based or self-report questionnaires, such as the IADL Lawton scale, by observing directly, in fact, an individual enacting an IADL, like making a phone call or managing money.

Farina et al. (2010) developed such direct performance-based measure of patients with dementia, e.g., the functional living skills assessment (FLSA) (Farina et al., 2010). This tool was conceived to detect functional impairment targeting high-order social abilities in everyday life and IADL by a clinician’s direct observation of the patient carrying out practical tasks or being verbally stimulated.

Nevertheless, those methods can be criticized as well, first, for being still strongly dependent on a human observer, and second, for removing the individual’s chosen routine and environmental cues that typically facilitate IADL. Finally, performance-based assessment can be often time-consuming (Sikkes et al., 2009) and represents a single evaluation data point compared with the multiple observations afforded by a questionnaire that comments on an individual’s overall behavior through the past weeks.

Information and communication technology (ICT) and, in particular, automatic video analyses of patients carrying out various IADL could be an innovative assessment method (Robert et al., 2013) to help overcome those limitations in reducing the inter/intra-rater variability due to human interpretation and increase ecological value by removing completely the human observer from the assessment site. Such techniques, and thus further, our proposed automatized video-based IADL assessment differs from these current tools by enabling the patients’ performances and actions to be captured in real time and real life situations and being accurately evaluated in order to provide the clinician with objective performance measures and a «second opinion»regarding the overall state of functionality of the patient.

In previous work, the use of such video sensor technology has already been demonstrated by Konig et al. (2014) by showing significant correlations between manually and automatically extracted parameters and neuropsychological test scores as well as high-accuracy rates for the detected activities (up to 89.47%). In a next step, we would like to investigate the use of video analyses for a completely automatized autonomy assessment based on the extracted video features.

In this line, the objective of this study is to investigate the use of ICT and, in particular, video analyses in clinical practice for the assessment of autonomy in IADL in healthy elderly MCI and AD patients by demonstrating an accurate automatized autonomy assessment based simply on automatically extracted video features from gait and IADL performances.

## Materials and Methods

### Study participants and clinical assessment

Participants aged 65 or older were recruited within the Dem@care protocol at the Nice Memory Research Center located at the Geriatric department of the University Hospital.

The study was approved by the local Nice ethics committee and only participants with the capacity to consent to the study were included. Each participant gave informed consent before the first assessment. It was a non-randomized study involving three diagnosis groups of participants.

The video data of 49 participants were exploitable from which 12 patients were diagnosed with AD, 23 patients diagnosed with MCI, and 14 healthy controls (HC). All diagnoses were made by a medical doctor from the Geriatric University Hospital.

For the AD group, the diagnosis was determined using the proposed diagnostic criteria from Dubois et al. (2007) requiring the presence of a progressive episodic memory impairment and biomarker evidence. For the MCI group, patients were diagnosed using the Petersen clinical criteria (Petersen et al., 1999) and only included with a mini-mental state examination (MMSE) (Folstein et al., 1975) score higher than 24. Subjects were not included if they had a history of head trauma with loss of consciousness, psychotic, or aberrant motor activity (tremor, rigidity, Parkinsonism) as defined by the movement disorder society unified Parkinson disease rating scale (Fahn and Elton, 1987) in order to control for any possible motor disorders influencing the ability to carry out IADL. Furthermore, participants with a MMSE score below 16 were excluded in order to avoid that the participant suffers from experiencing this assessment as a major failure.

Each participant underwent a standardized neuropsychological assessment with a psychologist. In addition, medical, clinical, and demographical information were collected. Global cognitive functioning was assessed using the MMSE (Folstein et al., 1975). Other cognitive functions were assessed, among others, with the FAB (Dubois et al., 2000) and the free and cued selective reminding test (Buschke, 1984; Grober and Buschke, 1987). Neuropsychiatric symptoms were assessed using the neuropsychiatric inventory (Cummings, 1997) and functional abilities were assessed using the IADL scale (IADL-E) (Lawton and Brody, 1969) during a clinical interview with the caregiver if there was one available.

### Clinical protocol

The clinical protocol asked the participants to undertake first a set of physical tasks (Scenario 1) and second a set of typical IADL (Scenario 2) followed by a free discussion period while being recorded by a set of sensors. Scenario 1 consisted of a single walking task and a dual task. The dual task involves walking while counting backwards from “305.” These tasks intend to assess kinematic parameters of the participant via gait analysis (e.g., duration, number of steps, cadence, stride length). Scenario 2, also called the “ecological assessment of IADL,” consisted of carrying out a set of daily living activities such as preparing a pillbox or writing a check within a timeframe of 15 min (see Table 1) followed by a short discussion. The defined activities were based on commonly used IADL questionnaires and represent at once activities with high- or low-cognitive demand [in accordance with the Bayer activities of daily living (ADL) scale] (Hindmarch et al., 1998; Erzigkeit et al., 2001). The protocol was conducted in an observation room located in the Nice Research Memory Center, which was equipped with everyday objects for use in ADLs and IADL, e.g., an armchair, a table, a tea corner, a television, a personal computer, and a library. Color-depth sensors (Kinect^®^, Microsoft^©^) were installed to capture the activity of the participants during the assessment. The aim of this protocol is an ecological assessment based on a “real time” performance that determines to which extent the participant could undertake independently a list of daily activities within a timeframe of 15 min. All assessments were performed at the same time of the day, between 2 and 3 p.m.

**Table 1:** **Design of ecological assessment**.

Part 1	Part 2
Guided activities (5 min)	Semi guided activities (30 min)
**TASK TO PERFORM**	
**Mono/dual directed tasks** – Walking – Counting backwards – Both walking and counting backwards **Vocal directed tasks** – Sentence repeating task – Articulation control task	**List of ADLs/IADL to organize and perform within 15 mn** – Watering plant – Preparing tea – Medication preparation – Managing finance (establishing account balance, writing a check) – Watching TV – Using phone (answering, calling) – Reading article and answering to questions
**CLINICAL TARGETS**	
Motor abilities: balance disorders Cognitive abilities: flexibility, shared attention, psychomotricity coordination, answer time to a stimulus, working memory	Cognitive abilities: flexibility, planification, shared attention, psychomotricity coordination, work memory, time estimation, answer time to a stimulus ADL/IADL performance

A clinician verified the performance of each participant in terms of the amount of initiated activities and correctly carried out activities as well as repetitions and omissions in order to define the quality of each task execution. Accordingly to this performance verification and based on previous work (Romdhane et al., 2012; Sacco et al., 2012; Konig et al., 2014), participants were grouped (independently from their diagnosis group) into either “good,” “intermediate,” or “poor” performer.

### Data collection and processing

**Figure 1:**
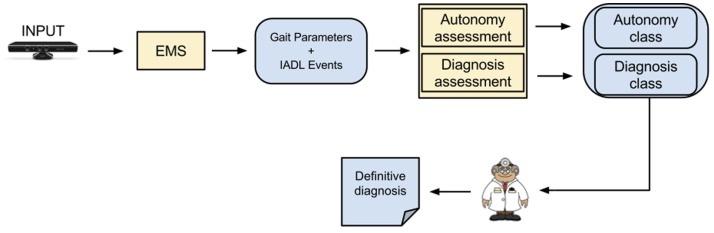
**System architecture; this figure shows the different steps from the system receiving the video input to the definitive clinical diagnosis**. The event monitoring system (EMS) consists of four modules that will lead to the correct assessment based on automatically extracted video features: people detection, people tracking, gait analysis and event recognition. The main outcome is based on the fusion of “Gait Parameters” and “Instrumental Activities of Daily Living Events,” which are processed with a feature selection method and a classifier for the autonomy and diagnosis assessment.

Participants had their activity recorded using a color-depth sensor placed close to the ceiling of the ecological room to maximize its coverage of the room. Recorded data were posteriorly analyzed by an event monitoring system (EMS, see Figure 1) to automatically extract fine- to coarse-grained information about patient’s performance (e.g., feet position, number of steps, the IADL carried out). Using the automatically extracted information, we estimate gait- and IADL-related parameters to describe the participant performance in the clinical protocol.

The estimated parameters were then used as input features for Naïve Bayes model to classify the participant’s performance into the autonomy and dementia classes investigated in this work. Targeted autonomy classes were good, intermediate, and poor; and targeted cognitive status classes were Alzheimer’s, MCI, and healthy.

### Event monitoring system

The EMS is composed of four main modules: people detection, people tracking, gait analysis, and event recognition. People detection step is performed by the background-subtraction algorithm proposed by Nghiem and Bremond (2014). The set of people detected in the scene is then tracked over the space and time by the algorithm of Chau et al. (2011). The output of these two modules is used as input for gait analysis and event recognition. The latter module is based on the work of Crispim-Junior et al. (2013), where a constraint-based ontology language is employed to model daily living activities in terms of posture, motion, and location patterns of the participant in the scene.

**Figure 2:**
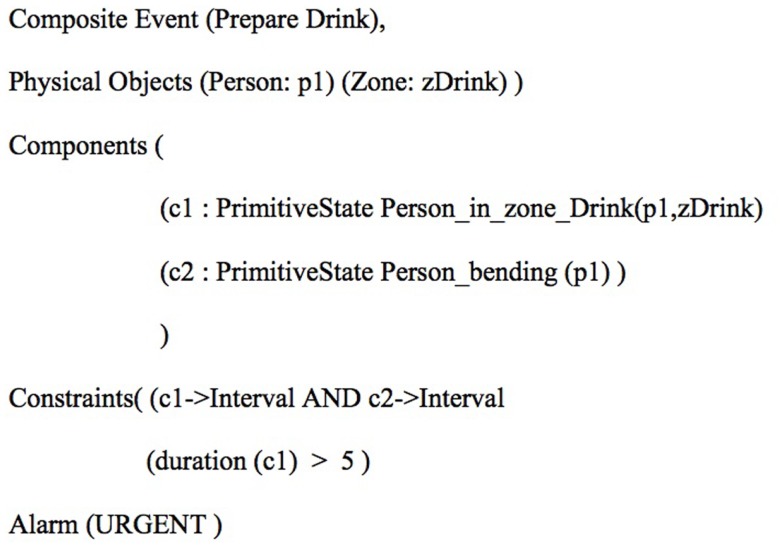
**Presents an example of event model for the recognition of preparing drink event following the ontology language**.

**Figure 3:**
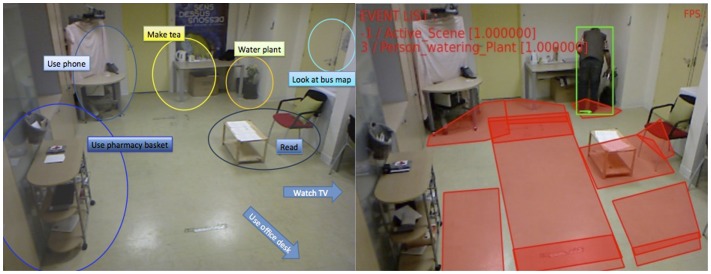
**Event recognition based on activity zones**. The left image presents the contextual zones used to describe the scene semantics. The right image presents an example of output of the automatic video monitoring system.

An IADL model is generally defined based on a set of physical objects (e.g., detected people, room furniture, and objects), a set of sub-events that model specific aspects of the targeted IADL, and constraints that establish rules sub-events and physical objects need to satisfy. Figure 2 presents an example of event model “Prepare Drink” using the ontology language. “Prepare Drink” event model is based on two sub-events (components): one event that verifies whether the person global position is located where the drinking objects are generally placed (named Person_in_zone_Drink), and a second sub-event verifying whether the person displays the posture “bending” (named Person_bending). Given that both components are recognized by the system, to satisfy the first constraint in the IADL model the person must be performing both sub-events at the same time (c1- > Interval AND c2- > Interval). The second constraint establishes that the first sub-event must have being performed for at least 2 s already. Once both constraints are satisfied, the event starts to be recognized by the EMS. For more details on IADL modeling, please refer to the work of Crispim-Junior et al. (2013). Figure 3 presents the monitored scene annotated with the semantic information used for event modeling and recognition. Left image displays the recognition of watering plant event.

The output of the EMS is the basis for the computation of the performance of the patients in the clinical protocol. From its output data (event report), we extract descriptors with different levels of granularity to appropriately describe the patient performance according to the complexity of the monitored activity. For gait analysis, we estimate fine-grained features like stride length, distance traveled, average speed, and cadence for the period of time of the physical task events (e.g., mono and dual task events). For the IADL, we compute their frequency and duration, and the number of times the patient missed or repeated them. Activity repetition and omission are calculated with respect to the number of times the participants are expected to perform an activity given the instructions they received at the beginning of the experiment. The ensemble of data automatically computed by the system constituted the behavioral profile (or performance assessment) of the monitored participant.

### Autonomy assessment and dementia diagnosis classification

Using the behavioral profile extracted by the EMS, two Naïve Bayes models were trained to classify participants into the targeted cognitive status and autonomy level classes according to their performance in the clinical protocol. To learn and validate the classifiers’ performance, we employed a 20-fold cross-validation scheme, where we partitioned the data set into 20 equal parts, and then performed model learning and validation 20 times. At each iteration, the cross-validation scheme retained one of the 20 folds for validation and used the other 19 parts for model learning. The reported model performance corresponds to the averaged performance of the models in the 20 validation folds.

To determine the best combination of parameters for dementia and autonomy classification, we performed feature subset selection based on best first search and Naïve Bayes classifier (Kohavi and John, 1997; Hall and Holmes, 2003). Using the feature selection method, we downsized the patient behavioral profile to the most relevant parameters for the classification of dementia and autonomy. The feature selection for both classification tasks (autonomy and dementia diagnosis) started with the same global feature set, algorithms were free to choose the input-parameters that maximized the performance of the task at hand.

We have selected the Naïve Bayes classifiers due to its probabilistic nature, which quantifies the pertinence of a participant’s performance for each class evaluated. Although this method assumes conditional independence among input parameters, an assumption that proves to be unrealistic for most practical application, it tends to perform reasonably well compared to more sophisticated methods, like support vector machines (John and Langley, 1995; Huang et al., 2003), with the advantage of having a much smaller running time and requiring very little training data (Matwin and Sazonova, 2012). All classification experiments were performed using WEKA platform (Hall et al., 2009). The implementation of Naïve Bayes in WEKA is based on the work of John and Langley (1995).

### Statistical analyses

In a separate step, next to the video data extraction analyses, the characteristics of all participants as well as the annotated performance results of the ecological assessment were analyzed in order to determine the different autonomy levels. Comparisons between the groups (e.g., HC subjects, MCI patients, and AD group and good performer, intermediate, and poor performer) were performed with Mann-Whitney tests for each outcome variable of the automatic video analyses. Differences were reported as significant if *p* < 0.05. Spearman’s correlations were further performed to determine the association between the extracted video parameters and established assessment tools, in particular, for executive functioning, e.g., the FAB.

## Results

### Population

Fourteen HC subjects (age = 74.1 ± 6.62), 23 MCI (age = 77.6 ± 6.17), and 12 AD subjects (age = 82.0 ± 8) were included. Tables 2 and 3 show the clinical and demographic data of the participants. Significant intergroup differences in demographic factors were found for age between MCI and AD subjects as well as between HC and AD subjects (*p* < 0.05). Further, significant differences were found between all groups for the MMSE score, with a mean of 28.4 (±1.1) for the HC group, 25.5 (±2.1) for the MCI group, and 22.67 ± 3.6 for the AD group (*p* < 0.05). Significant differences were found for FAB results between HC subjects with 16.3 (±1.1) and MCI subjects with 14 (±2.4), as well as between HC subjects and AD subjects with 12.33 (±3.1) (*p* < 0.05). The mean IADL scores did not differ between groups, with a mean IADL score of 7 (±1.2) for the HC group, 6.33 (±1.7) for the MCI group, and (6 ± 1.8) for the AD group.

**Table 2:** **Characteristics and group comparisons for HC, MCI, and AD subjects**.

Characteristics	All subject *N* = 49	Healthy control group *N* = 14	MCI group *N* = 23	AD group *N* = 12
Female, *n* (%)	26 (53.1%)	9 (64.3%)	10 (43.5%)	7 (58.33%)
Age, years mean ST	77.7 ± 7.3^†,‡^	74.1 ± 6.6	77.6 ± 6.2	82.0 ± 8
Level of education, *n* (%)
Unknown	0 (0%)	0 (0%)	0 (0%)	0 (0%)
No formal education	0 (0%)	0 (0%)	0 (0%)	0 (0%)
Elementary school	16 (32.6%)	2 (14.3%)	5 (21.7%)	9 (75%)
Middle school	9 (18.4%)	2 (14.3%)	6 (26.1%)	1 (8.3%)
High school	8 (16.3%)	4 (28.6%)	4 (17.4%)	0 (0%)
Post-secondary education	16 (32.6%)	6 (42.9%)	8 (34.8%)	2 (16.7%)
MMSE (mean ± SD)	25.6 ± 3.1*^,†,‡^	28.4 ± 1.1	25.5 ± 2.1	22.67 ± 3.6
FAB (mean ± SD)	14.25 ± 2.7*^,‡^	16.3 ± 1.1	14 ± 2.4	12.33 ± 3.1
FCSR test ± SD	39.2 ± 9.9*^,‡^	46.27 ± 1.9	38.19 ± 7.2	29.50 ± 16.7
IADL-E (mean ± SD)	6.4 ± 1.3	7 ± 1.2	6.33 ± 1.7	6 ± 1.8
NPI total (mean ± SD)	6.89 ± 8.1^†,‡^	3.54 ± 2.8	5.77 ± 7.1	12.6 ± 11
Ecological assessment results
Single task time (in s)	11.92 ± 3.1^†,‡^	10.79 ± 1.31	11.43 ± 2.97	14.36 ± 3.83
Dual task time	18.53 ± 8.19^†,‡^	14.79 ± 4.26	18.35 ± 8.78	23.25 ± 8.65
IADL
Activities initiated	9.16 ± 3.27*^,†,‡^	11.64 ± 1.15	9.39 ± 2.46	5.83 ± 3.61
Activities completed	6.65 ± 3.66*^,†,‡^	10.00 ± 1.47	6.57 ± 3.27	2.92 ± 2.27

*MCI, mild cognitive impairment; AD, Alzheimer’s disease; MMSE, mini mental state examination; FAB, frontal assessment battery; FCSR, free and cued selective reminding test; IADL-E, instrumental activities of daily living for elderly; NPI, neuropsychiatric inventory*.

All values represent means and SD (except *n*, gender, education and the classification results) *p < 0.05 for HC vs. MCI, †*p* < 0.05 for MCI vs. AD, ‡*p* < 0.05 for HC vs. AD.

*Group comparisons were made using Mann–Whitney *U* test (*p* < 0.05)*.

**Table 3:** **Intergroup comparison of scores and performance results from the ecological assessment (Mann–Whitney *U* test)**.

Comparison	Age *Z*/*p*	MMSE *Z*/*p*	FAB *Z*/*p*	FCSR *Z*/*p*	IADL *Z*/*p*	NPI *Z*/*p*	Single task	Dual task	AI	AC
HC vs. MCI	−1.695/0.090	−4.080/0.000	−3.024/0.002	−3.469/0.001	−1.603/0.109	−0.258/0.797	−0.286/0.775	−1.196/0.232	−3.067/0.002	−3.328/0.001
MCI vs. AD	−2.036/0.042	−2.432/0.015	−1.363/0.173	−1.024/0.306	−0.656/0.512	−2.228/0.026	−2.134/0.033	−2.003/0.045	−2.837/0.005	−3.093/0.002
HC vs. AD	0.023/−2.267	−4.261/0.000	−3.838/0.000	−2.654/0.008	−1.476/0.140	−2.433/0.015	−2.492/0.013	−2968/0.003	−4.121/0.000	−4.326/0.000

*MCI, mild cognitive impairment; AD, Alzheimer’s disease; MMSE; mini mental state examination; FAB, frontal assessment battery; FCSR, free and cued selective reminding test; IADL-E, instrumental activities of daily living for elderly; NPI, neuropsychiatric inventory; AI, activities initiated; AC, activities completed*.

**p* and *Z* values are presented for each group comparison*.

### Ecological assessment results

The participants performed differently on the IADL scenario in terms of initiated and successfully completed activities in accordance with their cognitive status. Tables 2 and 3 present results of the intergroup comparison of the performance results in the ecological assessment. Significant group differences were found for the single and dual task between MCI and AD (*p* < 0.05) and for HC and AD (*p* < 0.05). The amount of “activities initiated” and “activities completed” differed significantly between all three groups (*p* < 0.05).

The parameter “activities initiated” correlated significantly with neuropsychological test results, namely, the MMSE (*p* < 0.01), FAB score (*p* < 0.01), FCSR (*p* < 0.05), and the IADL-E score (*p* < 0.05). In the same line, the parameter “activity completed” correlated significantly with the test results, MMSE (*p* < 0.01), FAB score (*p* < 0.01), FCSR (*p* < 0.05), and the IADL-E score (*p* < 0.05). The obtained correlation analyses results are presented in Table 4. None of the extracted parameters correlated with the NPI total scores.

**Table 4:** **Correlation between IADL scenario performance and conventional cognitive assessments (Spearman’s correlation coefficient)**.

Video analyses data Spearman correlation coefficient (*r*)/*p*-values	MMSE	FAB	FCSR	NPI	IADL-E
Activities initiated	0.650**	0.519**	0.380*	−0.177	0.324*
	*p* = 0.000	*p* = 0.000	*p* = 0.019	*p* = 0.234	*p* = 0.030
Activities completed	0.685**	0.620**	0.356*	−0.266	0.334*
	*p* = 0.000	*p* = 0.000	*p* = 0.028	*p* = 0.071	*p* = 0.025

*p < 0.05 and **p < 0.01

**Figure 4:**
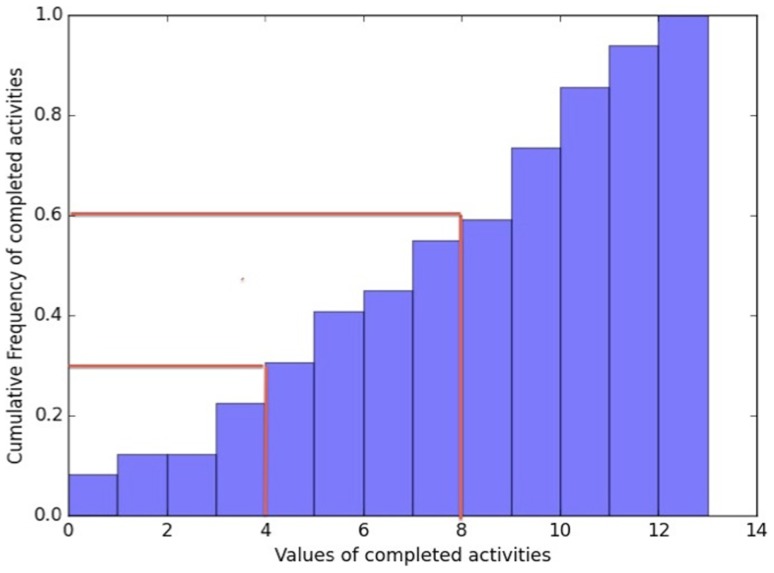
**Cumulative frequency curve of completed carried out activities**. The red lines indicate the cut-off scores between the autonomy classes which have been based on the analyses of the participant’s performances in terms of completely carried out activities, and on the cumulative frequencies of the completely carried out activities. These were divided in equal parts, as homogeneously as possible in terms of data coverage following the frequency curve.

After the performance analyses, the participants were classified based on their IADL performance. The cut-off scores between the classes have been based on the observation of the analyses of the participant’s performances in terms of completely carried out activities, and on the cumulative frequencies of the completely carried out activities. These were divided into equal parts as homogeneously as possible in terms of data coverage following the frequency curve as presented in Figure 4. This division into three equal classes resulted in the following cut-off scores.

From 13 to 8 completed activities was a good performance, meaning highly independent; from 7 to 4 completed activities was an intermediate performance; and below 4 completed activities was a poor performance, representing highly dependent in daily living activities. The grouping of the participants was done blinded from their diagnosis group in order to avoid classification bases, i.e., more likely to classify a HC as a “good” performer. A HC subject could sometimes show a mediocre IADL performance on the assessment and in turn a MCI subject could show a good IADL performance. Taking into consideration that the objective of the assessment was to stage autonomy levels and not necessarily disease progression, even though they are associated, it was important to make that differentiation. Table 5 shows the classification results based on the participants IADL scenario performances with their diagnosis group, as well as their average amount of completely carried out activities. Twenty-two participants from which 13 HC and 9 MCI subjects with an average of 10.04 correctly carried out activities were classified as good performer, 16 participants from which 1 HC, 10 MCI, and 5 AD subjects with an average of 5.5 correctly carried out activities were classified as intermediate performer and 11 participants from which 4 MCI and 7 AD patients with an average of 1.5 correctly carried out activities were classified as poor performer.

**Table 5:** **Ecological assessment results**.

	*N*	HC	MCI	AD	Activities completed (in mean ± SD)
Good performance	22	13	9	–	10.04 ± 1.4
Intermediate performance	16	1	10	5	5.5 ± 1.2
Poor performance	11	–	4	7	1.54 ± 1.4

### Validation of the event recognition system

Table 6 presents the results of the evaluation of the event video monitoring system (EMS) with respect to its precision at detecting correctly the events of the clinical protocol (scenario 1: single and dual task and scenario 2: the number of activities of daily living) annotated by domain experts while watching the experiment video.

**Table 6:** **Activity/event detection performance**.

Events	Recall (%)	Precision (%)
**Scenario 01**
Mono task	100.0	88.0
Dual task	100.0	98.0
**Scenario 02**
Searching bus line	58.0	62.5
Medication preparation	87.0	93.0
Watering plant	80.0	63.0
Reading article	60.0	88.0
Preparing drink	90.0	68.0
Talk on phone	89.0	89.0

Scenario 1, the single and dual task obtained the precision rates of 88 and 98%. From all proposed activities, “Medication preparation” was detected with the highest precision of 93% followed by “Using the phone” with 89% and “Reading an article” with 88%.

### Classification of participant cognitive status

Table 7 presents the classification results for autonomy assessment and dementia diagnosis. The classification procedure was intrinsically based on the features automatically extracted from the physical tasks and IADL performed by the participant during the clinical protocol. For comparison purposes, we have also learned two classifiers based only on behavioral data of the physical task or IADL-derived data. We hypothesized that combining the information from the two scenarios of the protocol increases the accuracy of the classification since they provide different but complementary information about a participant performance at daily living activities, e.g., motor and cognitive performances. For the three classifiers, the data set is the same and contains 49 patients in total. The overall activities were automatically detected with high sensitivity and precision results as previously described.

**Table 7:** **Classification results**.

Performance	Input data		
	Scenario 01	Scenario 02	Both scenarios
**Autonomy assessment**
Correctly classified instances	37 (75.5102%)	38 (77.551%)	41 (83.6735%)
Incorrectly classified instances	12 (24.4898%)	11 (22.449%)	8 (16.3265%)
**Diagnosis assessment**
Correctly classified instances	36 (73.4694%)	30 (61.2245%)	36 (73.4694%)
Incorrectly classified instances	13 (26.5306%)	19 (38.7755%)	13 (26.5306%)

In the Autonomy classification task the following features were employed:

- Single Task Total Duration,
- Single Task Gap Duration,
- Single Task Standard Deviation Steps,
- Dual Task Gap Duration,
- Dual Task Max Steps,
- Person using PharmacyBasket Frequency of Event (frequency),
- Person using PharmacyBasket Duration of Event (seconds).

For the Diagnosis classification, the set of features was:

- Age,
- Single Task Average Steps,
- Single Task Speed Average from Centroid Information,
- Dual Task Max Steps,
- Dual Task Min Steps,
- Person reading inChairReadingTable Duration of Event (Frames).

The classifier for dementia diagnosis task obtained an accuracy of 61.22% when using only features based on IADL (Scenario 2), and of 75.51% when just extracting features from physical tasks (Scenario 1). The accuracy rate increased up to 73.46% when combining features from both scenarios. However, the higher recognition rates were found for the classifier learned for autonomy classification; based on simply the automatically extracted video features from Scenario 2, 77.55% accuracy was obtained and 75% accuracy for Scenario 1. The highest accuracy rate of 83.67% was obtained when combining directed tasks and IADL.

## Discussion

The present study suggests that it is possible to assess autonomy in IADL functioning with the help of an EMS and that simply based on the extracted video features different autonomy levels can be classified highly accurately. The results obtained are significantly high not only for a correct assessment of autonomy but also cognitive status in terms of diagnosis. This means that “the proposed system” may become a very useful tool providing clinicians with diagnostic relevant information and improve autonomy assessment in AD or MCI patients in real time decreasing observer biases.

The results demonstrate further that gait analysis applied to IADL assessment may provide a reliable and precise methodology to assess patients functioning in daily life, which could be used at both diagnostic and rehabilitation levels. All extracted elements of the clinical protocol, the kinetic parameters from the single and dual tasks as well as the selected features from the IADL task are important and should be taken into consideration in the automatized analyses in order to assess and further predict accurately autonomy performances of patients. This means that in extractable gait features such as “single task standard deviation steps” and “dual task gap duration” lies relevant information about a patient’s capacity to perform IADL, and therefore, his or her autonomy level. These features added up to the automatically detected lengths and frequencies to carry out activities result in a highly accurate autonomy classification rate of almost 84%, allowing soon an almost fully automatized functional assessment in clinical practice.

The work of Gillain et al. (2009) illustrates in the same manner that it may be possible to determine different cognitive profiles, and hence autonomy levels, by the measurement of gait parameters. This confirms previous research findings that gait ability and cognitive functions are interrelated, and, in particular, executive functions and gait speed (Montero-Odasso et al., 2009; Beauchet et al., 2013; Doi et al., 2013, 2014). Gait impairment is already known to be a common characteristic of patients with MCI (Allan et al., 2005) and represents a risk factor for conversion to AD (Verghese et al., 2007; Buracchio et al., 2010). Therefore, changes in these motor functions may be useful in the early detection of dementia during preclinical stages and easily measurable by sensor technologies.

Furthermore, significant correlations were found between the parameters of initiated and completed activities and most neuropsychological test results, particularly with MMSE and FAB scores showing that group differences even with just a small sample size could be detected when using such techniques, and this when regular assessment tools such as the IADL-E questionnaire lacked sensitivity to detect these group differences.

Finally, high-single activity detection rates, up to 93% for the “Medication preparation” activity, could be achieved validating further the use of EMS for evaluation and monitoring purposes.

The study’s results were consistent with previous work where with a sensitivity of 85.31% and a precision of 75.90%, the overall activities were correctly automatically detected (Konig et al., 2015) although the present study was with a larger cohort and included AD patients as well.

Similar work, hence quantitative assessments of IADL performance, has been done using a different technique by Wadley et al. (2008) with the results that across timed IADL domains, MCI participants demonstrated accuracy comparable with cognitively normal participants but took significantly longer to complete the functional activities.

This suggests that slower speed in task execution could explain the differences found in the extracted features and thus, represents an important component and early marker of functional change already in the MCI patient, a component that would not be clearly identified using traditional measurements of daily function, but could be easily spotted using the quantitative and unbiased EMS data.

Likewise, Stucki et al. (2014) proved feasibility and reliability of a non-intrusive web-based sensor system for the recognition of ADL and the estimation of a patient’s self-dependency with high classification precision rates (up to 90%). Bang et al. (2008) used multiple sensor fusion (pressure sensors, passive infrared sensors, and worn accelerometers) for automatized ADL detection with achieved accuracy rates of up to 90%. Nevertheless, these studies were carried out with a very small group sample of healthy, and in average, younger participants.

Until now, the clinical assessment of functional changes in AD and MCI patients has traditionally relied on scales and questionnaires that are not always sensitive to the earliest functional changes. This leads to an important need to develop improved methods to measures these changes, ideally at the earliest stages. Therefore, recently research efforts have been placed on studies finding new innovative and more objective ways to measure functional and cognitive changes associated with AD (Vestal et al., 2006; Goldberg et al., 2010; López-de-Ipiña et al., 2012; Zola et al., 2013; Yakhia et al., 2014).

The main interest of the present study was to demonstrate the practical application of the use of such a video monitoring system in clinical practice. Now, once the systems’ use has been validated by significant correlation with neuropsychological test scores, particularly for executive functioning, and highly accurate detection rates, it can be employed as a supportive assessment tool within clinical routine check-ups also on a rehabilitation level and even move on to more naturalistic environments such as nursing homes.

The system’s extracted information can provide the clinician with direct measurements (see the list of features) indicating, once interpreted, a certain level of autonomy performance, as well as with information about possible underlying mechanisms caused by decline in certain cognitive functioning, namely, executive functions, which are highly associated (Marshall et al., 2011). This technique has the advantage of leaving out the clinician, who represents often in assessments a potential stress factor, completely from the evaluation site, and thus increasing ecological validity by leaving the patient alone in a more naturalistic “living-room alike” setting. The use of sensors for the measurement of behavioral patterns reduces important assessment biases often present in clinical practice and adds objectiveness to the assessment procedure.

The objective on a long term is to provide a stable system that allows monitoring patients and their autonomy at home over a longer period. The parameters validated within this study can serve as indicators for illness progression, decline in IADL performance and hence, executive functions detectable with the help of new technologies much earlier, before somebody in the family would notice and send the patient to a specialist.

The limitation of this study resides first in the age and education differences among the groups; the AD population was older than the other groups, and the HC and MCI group had higher levels of education. This can be partly explained by the recruitment process and that generally in clinical practice it is quite difficult to recruit young AD patients. However, age and education level differences could have had an impact on the IADL and gait performances and should therefore being taken into consideration.

Therefore, in future studies, it would be important to also focus on recruiting younger AD patients and participants with equal education levels in order to control for this variability. Second, the HC subjects were recruited through the Memory Center, which means that most of the HC participants came to the center with a memory complaint even though in their neuropsychological tests they performed within normal ranges. It has to be taken into consideration that those participants may not be completely healthy and suffer from a higher risk to convert to MCI than people that do not consult the center for a memory complaint (Jacinto et al., 2014).

It has to be further underlined that even if participants were alone during the IADL assessment, the simple fact of knowing that they were recorded could have had an impact on their stress level and thus, their performance.

Finally, it cannot be denied that the development of such a system and its analysis program was time-consuming and expensive. Engineers worked within the European FP7 Research Program Dem@care several years on improving the system’s efficacy and detection precision. However, once its usability in clinical practice has been further demonstrated by validation studies, its integration in routine assessment procedures is feasible; installation of such a system is affordable (Kinect camera and a computer) for Memory Clinics and analyses can be provided in real time. Nevertheless, more efforts in performance evaluation of such ICTs are needed to help the industry meet user needs and researchers in considering the available technologies for clinical practice. A solid economic model is a major issue: who will pay for assistive technology? Who will install and maintain ICTs at AD patients’ homes? The cost–effectiveness balance for assistive technology remains a matter of debate.

To conclude, according to the recently published review of Snyder et al. (2014), research efforts have launched large prevention trials in AD and these efforts have further clearly demonstrated a need for better and more accurate measures of cognitive and functional changes in people already in the earliest stages of AD. In the same line, the US Food and Drug Administration elevated the importance of cognitive and functional assessments in early stage clinical trials by proposing that even in the pre-symptomatic stages of the disease, approval will be contingent on demonstrating clinical meaningfulness.

Similiarly, Laske et al. (2014) argued that there is an increasing need for additional non-invasive and/or cost-effective tools, allowing identification of subjects in the preclinical or early clinical stages of AD who could be suitable for further cognitive evaluation and dementia diagnostics. Once examined in ongoing large trials, the implementation of such tools may facilitate early, and potentially more effective therapeutic and preventative strategies, for AD.

All this points out, the need for improved cognitive and functional outcome measures for clinical studies of participants with preclinical AD and those diagnosed with MCI due to AD. With our study, we propose a new method of measuring objectively and accurately functional decline in patients from the earliest stages on with the support of the vision sensor technologies; a reliable method that could potentially, once validated through larger scale cohort studies, serve within clinical trial of new drug interventions as an endpoint measure to prove their effects on ADL function. Finally, the use of such systems could facilitate and support aging-in-place and improve medical care in general for these patients.

## Conflict of Interest Statement

The authors declare that the research was conducted in the absence of any commercial or financial relationships that could be constructed as a potential conflict of interest.
